# Strain Measurements within Fibreboard. Part III: Analyzing the Process Zone at the Crack Tip of Medium Density Fiberboards (MDF) Double Cantilever I-Beam Specimens

**DOI:** 10.3390/ma5112190

**Published:** 2012-11-07

**Authors:** Jörn Rathke, Ulrich Müller, Johannes Konnerth, Gerhard Sinn

**Affiliations:** 1 Wood K plus—Competence Centre for Wood Composites and Wood Chemistry, Altenberger Straße 69, Linz 4040, Austria; E-Mail: ulrich.mueller@kplus-wood.at; 2 Department of Material Sciences and Process Engineering, Institute of Wood Technology and Renewable Resources, BOKU—University of Natural Resources and Life Sciences, Konrad Lorenzstraße 24, Tulln an der Donau 3430, Austria; E-Mail: johannes.konnerth@boku.ac.at; 3 Department of Material Sciences and Process Engineering, Institute of Physics and Material Science, BOKU—University of Natural Resources and Life Sciences, Peter Jordan Straße 82, Vienna 1190, Austria; E-Mail: gerhard.sinn@boku.ac.at

**Keywords:** double cantilever I-beam test, electronic speckle pattern interferometry, fracture mechanics, fracture energy, medium density fiber board

## Abstract

This paper is the third part of a study dealing with the mechanical and fracture mechanical characterization of Medium Density Fiberboards (MDF). In the first part, an analysis of internal bond strength testing was performed and in the second part MDF was analyzed by means of the wedge splitting experiment; this part deals with the double cantilever I beam test, which is designed for measuring the fracture energy as well as stress intensity factor in Mode I. For a comparison of isotropic and orthotropic material behavior, finite element modeling was performed. In addition to the calculation of fracture energy the stress intensity factor was analyzed by means of finite elements simulation and calculation. In order to analyze strain deformations and the process zone, electronic speckle pattern interferometry measurements were performed. The results revealed an elongated process zone and lower results for K_IC_ if compared to the wedge splitting experiment. The G_f_ numbers are higher compared to the wedge splitting results and can be explained by the thicker process zone formed during the crack propagation. The process zone width on its part is influenced by the stiff reinforcements and yields a similar crack surface as with the internal bond test.

## 1. Introduction

The mechanical characterization of wood based panels includes, according to European standards [1,2,3] tensile, bending, and internal bond strength tests. The testing of bending and internal bond strength is used for quality assurance purposes in the entire production chain. For the characterization of the core layer, however, several problems occur when following the conventional testing procedures. The testing of bending strength reflects a combination of tensile, compression and shear strength. Internal bond strength testing (IB) is a simple test where specimens are adhesively bonded to braces and tested in tension perpendicular to the panel plane. This testing procedure includes a high number of effects which can bias the IB values to a large degree, such as for instance overlap of the adhesive at the edges, incomplete bonding of the specimen to the braces, varying bond line thickness and inhomogeneous transfer of stresses from the braces to the specimen [4]. The latter induces stress concentrations within the specimen, which in turn influences the IB value measured. This phenomenon is more precisely described in part 1 of this trilogy [4]. Nevertheless, the IB test provides only one mechanical parameter, which refers to internal bond strength. No further information, such as for instance Young’s modulus or the fracture energy, can be derived from this procedure.

Fracture concepts promise a higher yield of information concerning material characteristics and the separation process. There are two different basic concepts in fracture mechanics, *i.e.*, linear elastic fracture mechanics (LEFM) and non-linear elastic fracture mechanics (NLEFM). In terms of wood based panels, LEFM is widely used [5,6,7,8] but the basic assumption of a linear elastic material is only partially fulfilled. Therefore, the use of LEFM for wood and wood based materials is limited because crack propagation within wood and wood based materials includes a fracture process zone (FPZ) in which an increasing number of micro cracks happens before macro-cracking can be seen [9]. Additionally fiber bridging may hinder crack growth. Still, LEFM is used as it allows a comparative value to materials such as for instance metals and concrete. Anyway, the described drawbacks can be overcome by the NLEFM analysis.

One test set-up for wood and wood based panels with NLEFM is the wedge splitting experiment [10]. This testing procedure has been used for several test setups in the field of fracturing processes of wood based panels [5,11,12,13,14]. The fracture energy is thereby determined by integration of the area below the load displacement curve.

For the process optimization of the wood based panel production, the characterization of the core layer is of outstanding importance. The core layer is most often used to adjust the density of panels e.g., when lightweight materials are desired [15,16]. The desired core layer properties significantly influence the pressing process *i.e.*, production speed, temperature and pressing profile. Therefore, the internal bond strength of the core layer is the most important factor limiting the production speed because the resin has to be cured to a certain degree before the press can be opened again [17,18]. Several different approaches to characterize the core layer have been proposed in earlier works. One of these approaches was followed by Ehart *et al.* [13], who used the wedge splitting set-up. Here, the crack propagation in particleboard perpendicularly and parallel to the board plane were analyzed. The performed tests showed that testing the core layer of thin particleboards is not possible without supporting the surface layers. Another test analyzing the core layer of medium density fiberboards (MDF) was performed by Matsumoto and Nairn [19]. In their experiment, extended compact tension (CT) specimens were used and the crack initiation energy was calculated. Double cantilever beam (DCB) specimens were used by Yoshihara [20] to analyze the fiber bridging in the core layer of MDF.

In the present work the Double Cantilever I-Beam (DCIB) test [21,22,23] is discussed as an alternative method for the testing of wood based panels. The described testing procedure was developed as a fast set-up for quality control of the core layer of wood based panels and consists of two stiff steel braces, which are glued to the face layers. Using fracture mechanical test setups, the middle layer of wood based panels cannot be tested easily without reinforcements due to excessive bending and resulting damage of the face layers thereof. From a theoretical point of view the composite specimen (wood based panel and steel) is not desirable as the measured fracture toughness might not result in the critical plane strain stress intensity factor but it depends on the specimen composition. Nevertheless it is possible to determine an approximate critical stress intensity factor, which allows a comparison of different materials. This approach, for instance, was demonstrated to be useful for the analysis of the influence of the raw material [21], the particle type and the resin content [22] on wood based panels.

The aim of this study is (1) to compare the well-known wedge splitting set-up [5] with the DCIB set-up [21,22,23] based on the example of MDF data from part 1 [4] and 2 [24] of the trilogy. Stress intensity factor and specific fracture energy will be evaluated. For the determination of the stress intensity factor finite elements calculations (2) were performed. In the part of this study (3), the stress and strain distributions on the specimen surface under load analyzed by means of electronic laser speckle interferometry (ESPI). ESPI experiments were performed to study the FPZ developed by loading the sample. Measuring deformations in the region of the crack tip should allow estimating the size of the FPZ and area where micro cracks occurred. Based on this data, (4) the crack length can be determined by simple length measurements.

## 2. Experimental Procedure

In this paper the newly developed Double Cantilever I-Beam test was used for the analysis of medium density fiberboards (MDF) with a thickness of 38 mm and a density of 711 ± 6 kg/m^3^. Before testing the specimens were stored in standard climate (20 °C/65%) until equilibrium moisture content was reached. The specific fracture energy was calculated by means of integral calculus of the load displacement curve and the stress intensity factors were calculated using FE simulation. For the analysis of the FE simulation and for the determination of the crack length, ESPI measurements were performed. In total six specimens were analyzed for specific fracture energy and stress intensity factor calculation and three specimens were analyzed for ESPI measurements.

### 2.1. Specific Fracture Energy

**Figure 1:**
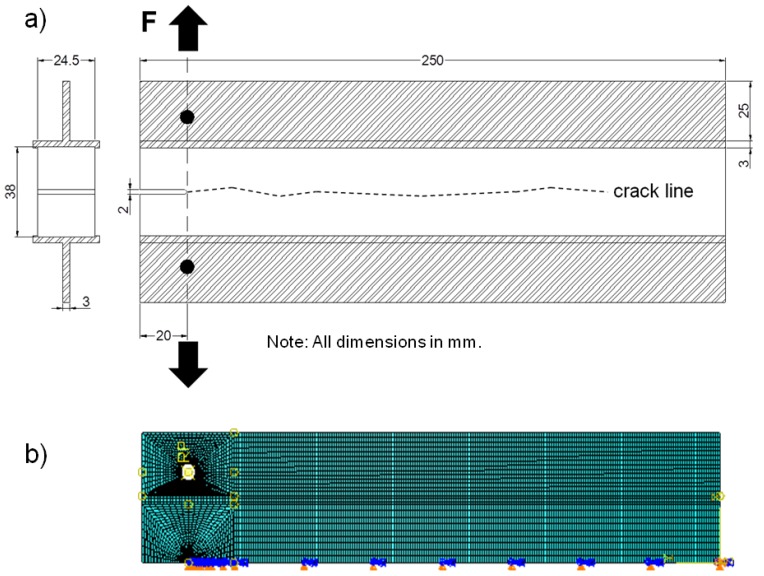
(**a**) Sketch of the Double Cantilever I-Beam specimen; (**b**) half of the symmetrical finite element model representing the specimen and showing the boundary conditions.

The DCIB specimens had a length of 250 mm and a width of 24.5 mm (Figure 1a). The density of each specimen was determined by means of dimensional and gravimetric measurements. A notch of 20 mm depth was then sawn into the middle layer parallel to the panel surfaces using a band saw (saw kerf-thickness 2 mm). Finally, two metallic braces were glued to the face layer surfaces with a fast-curing cyano-acrylate adhesive (Loctite 431, Henkel). The specimen shape was designed such in a way, that the loading points are in-line with the tip of the kerf. As reinforcements of the face layers, metallic T-beams were used.

The testing of fracture energy introduces a tensile load perpendicularly to the middle layer area, which leads to fracture in Mode I. The specimens were clamped into fasteners with pins. Directly before testing, a thin initial kerf was cut into the ground of the notch, using a sharp razor blade. For testing, a load was applied at the notched end of the specimen. A cross head speed of 1 mm/min was chosen. After reaching a force drop of 50% of the maximum load, a progressive increase of the cross head speed up to 10 mm/min was applied. With this test setup, the maximum load is reached within 60 ± 30 s. The test was stopped after a maximum displacement of 50 mm or a remaining force of 5 N.

These settings guaranteed a testing period of three minutes as a maximum duration. The fracture energy was calculated by an integration of the area below the load-displacement curve. The result reflects the fracture work required to split a specimen into two halves. According to Sinn *et al.* [12] and Rathke *et al.* [22], the specific fracture energy *G*_f_ is the energy applied in the stable or quasi-stable fracture of a notched specimen averaged over the fracture area. Relating the separation area to the fracture work, the specific fracture energy (J/m²) can be calculated according to Equation (1):

(1) $$G_{f}=\frac{1}{(L-a)B}\int_{0}^{z_{\max}}Fdz$$

where *F* is the applied force, *z* is the displacement at the loading point, *a* is the initial crack length and *L* and *B* are the total length and the width of the specimen, respectively.

### 2.2. Finite Element Calculations

Despite the fact that wood [5,8] and wood based panels [13] show non linear characteristics, which refers to the formation of a large process zone and fiber bridging behind the crack tip [25], most publications use the concept of linear elastic fracture mechanics [6,7,12,13,24]. In most cases the determined stress intensity factors K_IC_ are calculated from single edge notched bending specimen. This assumes isotropic material characteristics. A comparison of the isotropic and orthotropic fracture toughness was performed for the wedge splitting experiment by Schachner *et al.* [25]. It was found that the RL orientation in spruce wood, with loading perpendicular to the fiber orientation, shows the smallest difference between orthotropic and isotropic calculations, which reflects a similar FPZ as with the DCIB test setup. To enable a comparison of orthotropic and isotropic stress and strain behavior of DCIB specimen when load is applied, finite element simulations were performed, using ABAQUS® software. The initial values of the material parameters were based on own experiments, as well as tension strength, bending strength, modulus of elasticity and internal bond strength data gained from Kollmann *et al.* [26] and Niemz [27]. The finite element model with symmetry boundary conditions is shown in Figure 1b. The modeled specimen was loaded with a localized vertical force at the reference point RP of the pin. The pin itself was modeled as a rigid body.

### 2.3. Stress Intensity Factor

Tabulated formulas for stress intensity factors are based on the assumptions of isotropic materials and simple geometries; therefore it was necessary to perform FE simulations of the composite DCIB specimen. Material tests were performed for the determination of the critical stress intensity factor K_IC_ under Mode I considering composite DCIB-specimen. The data gained was used for a symmetrical finite element simulation of one half of the specimen. The simulation problem was reduced to a two- dimensional, plane strain model. Isotropic and linear material properties were assumed. The (half) crack tip was simulated with 30 collapsed 8-node biquadratic plane strain elements with mid-side nodes placed at ¼ of the distance along the element side to create quarter-point elements representing $1/\sqrt{r}$ stress singularity. The stress intensity factor algorithm from ABAQUS® was used for the calculation. To this end a number of simulations with an adjustable isotropic modulus of elasticity of the board, were performed and the stress intensity factor, normalized by the loading force, was determined as a function of the modulus of elasticity, respectively the initial slope. Equation (2) was finally derived by fitting the normalized stress intensity factors from simulations as a function of the initial slopes and multiplying the result with the load. The relative error between the FE-simulation and Equation (2) is less than 0.5% for $3.165<\frac{k_{init}}{B}\cdot \left(\frac{{\text{mm}}^{\text{2}}}{\text{N}}\right)<1100$.

(2) $$K_{IC}=F_{\max}\left[6.568\cdot 10^{-5}+2.082\cdot 10^{-10}\frac{k_{init}}{B}-1,498\cdot 10^{-10}\cdot {\left(\frac{k_{init}}{B}\right)}^{2}+5.253\cdot 10^{-14}\cdot {\left(\frac{k_{init}}{B}\right)}^{3}\right]$$

In this equation *F_max_* is the peak load, *k_init_* is the slope in (N/mm) of the linear regression line calculated from load displacement data in the range of 25% to 50% of *F_max,_* and *B* stands for the specimen width in millimeter.

### 2.4. Speckle Measurement

**Figure 2:**
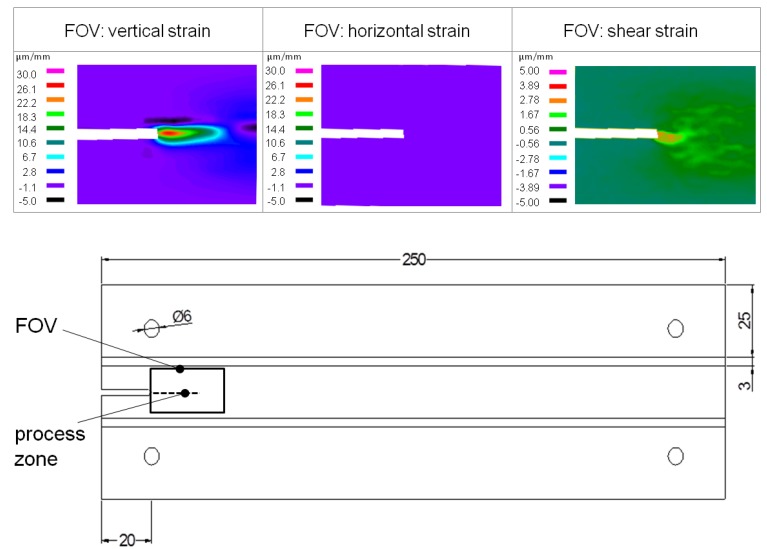
Representative results of electronic laser speckle measurements showing the in-plane strain distribution $\varepsilon_{yy}$ (μm/mm) in vertical orientation (left), $\varepsilon_{xx}$ (μm/mm) in horizontal orientation (middle) and the shear strain ½ ($\varepsilon_{xy}$ + $\varepsilon_{yx}$) (μm/mm) (right)

For the analysis of the strain field and to evaluate the information gained form the FE simulation, measurements using electronic laser speckle pattern interferometry (ESPI) were performed. Three specimens were used for the analysis of the crack length and the formation of the process zone. The deformations of the samples were measured on one side of the DCIB specimen. A proper field of view (FOV) for the ESPI measurement was chosen to observe the whole process zone (see Figure 2).

As described in Rathke *et al.* [4] and Sinn *et al.* [24], the deformation of the sample surface causes a change in the phase difference and therefore a new speckle pattern. To calculate an image with the typical fringe pattern, each image pattern is subtracted from the previous one [28]. The analysis was performed according to Rathke *et al.* [4]. A more precise description of the Electronic Laser Speckle Interferometry measurements is given in part 1 of the trilogy [4] as well as in other literature [28,29,30,31,32].

## 3. Results and Discussion

### 3.1. Specific Fracture Energy

Specific fracture energy tests were performed to analyze six specimens of medium density fiber board with a thickness of 38 mm and an average density of 711 ± 6 kg/m^3^. The specific fracture energy, determined by means of the Double Cantilever I-Beam test, was shown to be 161.12 ± 13.27 J/m^2^ with a corresponding coefficient of variation of 8.23%. The coefficient of variation is significantly lower than that of the internal bond strength determined in part 1 of the trilogy [4], where the coefficient of variation was 37.2%. The data for specific fracture energy gained by means of the wedge splitting experiment in part 2 of the trilogy [24] reveals only approx. 31.8% (45.2 ± 14.4 J/m^2^) of the numbers which were generated with the DCIB experiment. It seems probable that a size effect which is described in Ehart *et al.* [13] is responsible for a certain contribution to this difference: both specimen geometries had the same width, but while the DCIB specimen were 250 mm long, the specimens for the wedge splitting experiment had a length of only 125 mm and a ligament length of 90 mm. To estimate the magnitude of the size effect wedge splitting specimens were prepared and reinforced by means of DCIB load block elements. The resulting wedge splitting specimens are similar stiff as the DCIB specimens for similar crack lengths. The ligament length was set to 90 mm as with the test set-up in Sinn *et al.* [24] and a total of six specimens were tested. The results revealed specific fracture energy numbers of 114 ± 9 J/m^2^ which is much closer to the results gained by means of DCIB testing than the results from Sinn *et al.* [24] measured with the wedge splitting set-up simply reinforced by steel plates *G*_f_ = 45.2 J/m^2^ ± 14.4 J/m^2^. It might be concluded, that the reinforcement has a strong influence on the fracture process and therefore on the determination of the specific fracture energy. Additionally it might be concluded that there is active a significant size effect.

To ensure the correctness of the measured *G*_f_ numbers derived with the DCIB experiments, the data was compared with numbers found from literature. To this end, the specific fracture energy was calculated from the fracture toughness (G_c_) numbers provided by Matsumoto and Nairn [19]. Matsumoto and Nairn used extended CT specimens to analyze the fracture toughness by means of elastic fracture determination. A G_c_ number of 48.4 J/m^2^ a strength σ_c_ of 0.1 MPa and a slope of the rising R-curve of 303 J/m^3^ were determined for specimens in z-orientation. Crack-plane interference due to fiber bridging means that the material cannot be unloaded back to its original position in the specimen. Therefore, a residual displacement can persist even if the fracture process is performed under elastic conditions. The described difficulty forced Matsumoto and Nairn [33] to use a revised R-curve analysis without unloading the specimen to describe the fracturing process. For this procedure, the cumulative released energy per unit thickness *U(x)* and the crack length *a(x)* are measured as functions of displacement *x*. The fracture extension resistance curves (R curves) are determined by numerically differentiating the energy U(x(a)) with respect to the crack length *a* (Equation (3)).

(3) $$R=\frac{dU(a)}{da}=\frac{dU\left(x\right)}{dx\left(a\right)}\frac{dx\left(a\right)}{da}$$

The extended CT specimens had a total length of 127 mm (5 inch) and a ligament length of 77.5 mm. To perform a comparison of the specific fracture energy using the extended CT specimens and the DCIB specimen geometry, Equation (4) was generated using the definitions of the involved parameters.

(4) $${\text{G}}_{\text{f}}=\frac{1}{\left(L-a\right)}\int_{0}^{L-a}dU=\frac{1}{\left(L-a\right)}\int_{0}^{L-a}Rda=\frac{1}{\left(L-a\right)}\int_{0}^{L-a}(G_{c}+Slope\;a)da=G_{c}+\frac{1}{2}Slope(L-a)$$

Calculating the specific fracture energy from fracture toughness numbers given by Matsumoto and Nairn [19] yields a G_f_ value of 60.14 J/m^2^ (for the ligament length of 77.5 mm), which is 37% of the mean value determined by means of DCIB testing procedure (161 J/m^2^). One possible explanation of this discrepancy might be found in the ligament length, which is only one third of the length analyzed with the DCIB specimen geometry and another in the steel reinforcements used for the DCIB tests. Furthermore Matsumoto and Nairn [19] used a material with similar density but deviant thickness (19.05 mm). The thickness was therefore 20% less than that of the DCIB specimens.

Anyway, due to the test results, of the symbiosis test setup of DCIB and wedge splitting experiment a size effect of the experiments is not able to explain the entire differences. Part of the differences might be explained by the reinforcement of the face layers which influences the stress field at the crack tip.

## 3.2. FE Simulation

**Figure 3:**
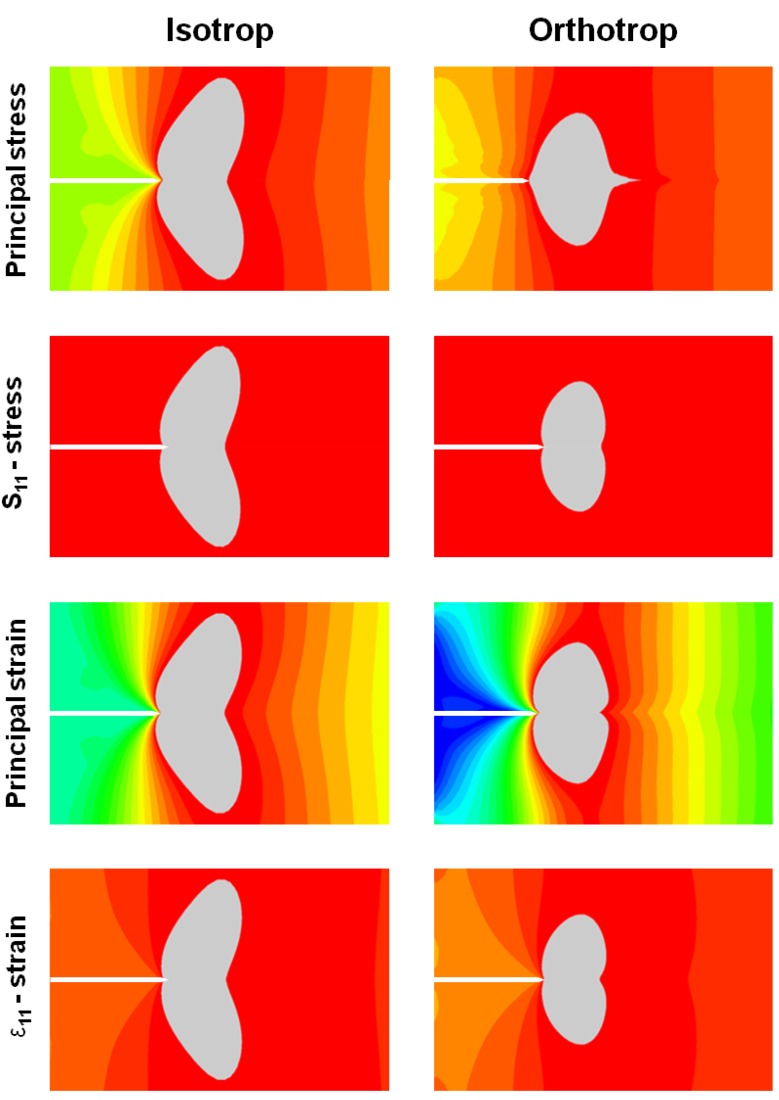
Comparison of isotropic and orthotropic FE simulation.

In Figure 3, a comparison of isotropic and orthotropic finite element simulations for the DCIB specimen geometry is presented. The main task of this simulation was to analyze the length and shape of the process zone. The grey regions show the process zone were $\sigma \geq \sigma_{ib}=0.51$ MPa or $\varepsilon \geq \frac{\sigma_{ib}}{E}$; within this region the real material would fail. The internal bond strength determined in part 1 of this trilogy [4] is used for convenience, as no other strength parameters are available.

**Figure 4:**
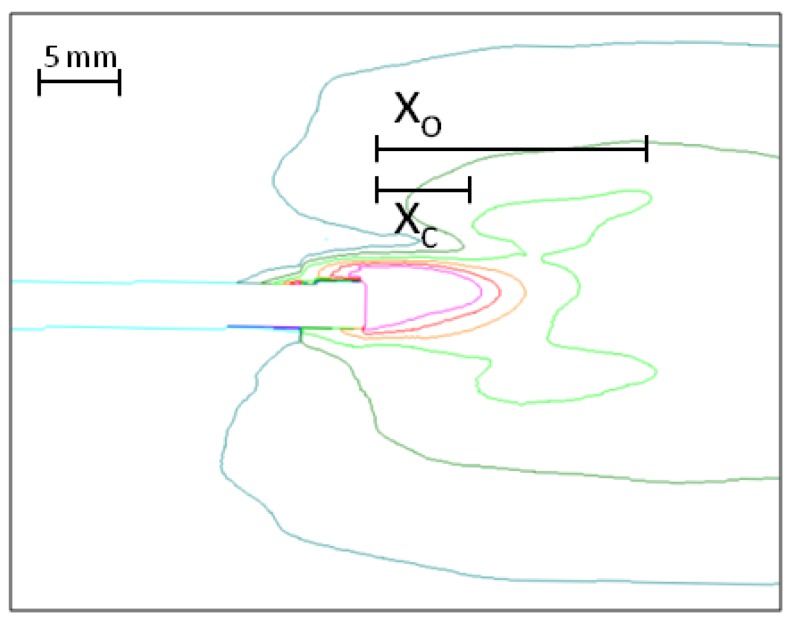
Crack length determination by means of ESPI measurements.

The figure shows four options of data output in the region of the Field of View (FOV), described in Figure 4. First the distribution of the principal stress is shown, then the stress S_11_ in vertical direction, the principal strain and finally ε_11_ strain. Besides the results for the first principal stresses, there are apparently no differences in the length of the process zone. Another effect, which can be seen from the equally scaled pictures generated by means of FE simulations, is the lesser expansion in vertical direction of the affected area for the orthotropic calculations. While the isotropic simulation reveals a stretched zone in tension direction, the orthotropic simulation shows a truncated region.

Material parameters used for and as a result of the simulations are summarized in Table 1. Whereas all parameters beside E_11_ were fixed, E_11_ is the result of an adaptation process to reproduce the experimental initial slope.

**Table 1:** Elastic parameters used for simulations.

	E_11_/MPa	E_22_/MPa	E_33_/MPa	G_12_/MPa	G_13_/MPa	G_23_/MPa	ν_12_	ν_13_	ν_23_
orthotropic	270	4000	4000	100	100	600	0.01	0.01	0.11
isotropic	154						0.1		

### 3.3. Stress Intensity Factor

Stress intensity factors were calculated with Equation 1 using experimental initial slope and maximum load as input values. The data revealed a mean value of 0.07 ± 0.01 MPam^0.5^ with a coefficient of variation of 12.2%. This data is a bit higher than the numbers found in Rathke *et al.* [21], where the K_IC_ numbers revealed—depending on the orientation of the specimen to the production direction—values of 0.04 MPam^0.5^ (longitudinal) and 0.05 MPam^0.5^ (orthogonal). In part 2 [24], stress intensity factor calculation revealed numbers of 0.1 MPam^0.5^—with a coefficient of variation of 13.5% using the wedge splitting experiment. While the coefficient of variation for the DCIB test is a bit lower than for the wedge splitting experiment, the K_IC_ numbers of the DCIB test setup are about 30% lower. The difference here can probably be explained by a longer process zone of the DCIB specimen geometry in contrast to the wedge splitting experiment. Disregarding the bigger plastic zone size in the calculation of *K_IC_* as a longer, effective crack length *a_eff_* might explain the lower values for *K_IC_* found for the DCIB specimens. Therefore the stress intensity factor determined by the DCIB test set-up might not be considered as a real material property and therefore as the critical plane strain toughness. The stress intensity factor shows to be dependent on the specimen geometry and especially on the stiffness of the steel reinforcements. Nevertheless one of the main goals, when the specimen was developed, was to create a simple, yet objective test setup for testing the core layers of plate materials. This goal is unaffected, because relative comparisons are still possible.

In literature only a few experiments can be found which analyze the fracture toughness of MDF. Niemz *et al.* [6,7] used the CT specimens according to ASTM E 399 to analyze the stress intensity factor. The specimens were oriented parallel to the board plane and yielded therefore a mixture of face layer and core layer properties with a K_IC_ of 1.81 ± 0.33 MPam^0.5^ (CV 18.2%) for a density of 710 kg/m^3^ (20 °C/ 65% RH) and numbers in a range of 0.36 ± 0.03 MPam^0.5^ (8.3% CV) to 1.29 ± 0.06 MPam^0.5^ (CV 4.7%) with a density of 500 kg/m^3^, depending on the equilibrium moisture content (21.4% to 3.5%). The differences between the data calculated for the DCIB test set-up and the data from Niemz *et al.* [6,7] can be explained by the specimen orientation which reflects a combination of face layer and core layer in contrast to own measurements were only the core layer was tested by means of the DCIB test set-up. In part two of this trial [24] the stress intensity factor were calculated from the wedge splitting experiments on MDF. The results for MDF show a K_IC_ of 0.11 ± 0.02 MPam^0.5^, which is also a bit higher than data gained from our own experiment.

### 3.4. ESPI Measurements

For the measurement a Dantec Ettemeyer Q300 (Dantec Ettemeyer, Ulm, Germany) ESPI system was mounted on the testing machine. The high sensitivity of the system requires a constant control of the field of view (FOV) while the experiment is performed. The FOV was placed in the specimen section of the initial notch and the fracture zone (see Figure 2). The optical axis of the ESPI system underwent the same movements during the testing procedure as the center part of the specimen. The working distance between the optical system and the specimen surface was 357 mm and a total area of 45.7 mm × 36.2 mm (FOV) was observed.

The results of ESPI measurements for DCIB strain and shear strain measurements are presented in Figure 2. The strain maps are representative examples for the three DCIB specimens tested and allow a determination of the deformations of the specimen under load in Mode I.

The vertical strain map shows a zone of positive strain concentration in the middle of the core layer. The zone is elongated and represents the crack propagation zone. The crack propagation zone can be subdivided into a zone with high strain (18 to 30 µm/mm) and a zone with low strain (5 to 18 µm/mm). A zone with slightly negative strain values can be seen directly above the crack propagation zone and in front of it. A negative strain generally implies a compression of the particles. For this test configuration compression above the crack propagation zone should not occur. Anyway, the negative strain measured is negligible small compared to the positive strain. Random noise during the measurement (e.g., movement and deformation of single fibers on the surface of the specimen) is thought to be responsible for small negative values observe. In contrast to the high variety of strain in the vertical direction, no strain at all is found in the horizontal direction. For the analysis of the shear strain, the map of vertical displacements gained from ESPI experiments was differentiated in horizontal direction (ε_yx_) and the map of horizontal displacements was differentiated in vertical direction (ε_xy_). The shear deformation was calculated as follows: ½ (ε_yx_ + ε_xy_). It can be seen that a hot spot of shear strain is directly in line with the crack propagation zone. The supposition of micro cracking in the process zone is also described by Ehart *et al.* [13] for the fracture process of particleboard by means of wedge splitting experiment, and for MDF by Matsumoto and Nairn [19] for extended CT specimens. While Ehart *et al.* [13] performed a description of the fracture process by means of visual inspection, Matsumoto and Nairn [19] analyzed the bridging effects by means of Digital Image Correlation (DIC). However, ESPI measurements reveal a higher yield of results as not only the crack length, but also the size of the strain and the shear strain areas can be detected.

### 3.5. Crack Length Determination

Using standard measurement devices, the determination of the crack length is hardly possible with materials that display a high rate of fiber bridging, as the exact position of the crack is impossible to measure. Digital Image Correlation (DIC) might be one system for the analysis of fracturing process, anyway the higher resolution and accuracy of the ESPI system predestines this procedure for the measurement in this test. The ESPI measurement device promises to overcome the difficulty of fiber bridging as well as micro cracking and allows an analysis of the process zone.

When load is applied to the specimen, the process zone develops. In this trial the specimens were tested in the weak direction of the board, *i.e.*, perpendicular to the board plane, which leads to crack propagation in the core layer parallel to the board faces. Figure 4 shows a picture of ESPI profile strain measurement, which represents the crack process zone. It is evident from the figure that the process zone of MDF can be divided into two regions, a region of high strain up to the actual crack tip at *x_c_* and a less strained region in front of the crack tip. Both zones together compose the total process zone with the length *x_o_*.

Figure 4 shows a representative image of the ESPI measurements with the dimensions 36.23 mm height and 43.65 mm length. In the image displaying the ESPI measurement, the red/yellow colored lines represent the high strained region (*x_c_*) and represent deformations from 18 to 30 µm/mm. This region is highly damaged and load is carried by fibers bridging the crack faces.

Using the IB strength from part 1 of this article series, $\sigma_{IB}=0.51$ MPa and the modulus reversely calculated from FE simulations the strain at failure is about $\varepsilon_{f}=4.33\;\frac{\mu \text{m}}{\text{mm}}$ (orange line in FOV of ESPI measurement). Dark green and turquoise show the strain distribution in the region of 5 to 18 µm/mm and represents the total process zone length (*x_o_*). The measured crack length data is presented in Table 2.

**Table 2:** Crack length results.

Specimen	High strain (*x_c_*) (mm)	Low strain zone length (mm)	Total length (*x_o_*) (mm)
1	7.56	9.20	16.76
2	6.76	8.72	15.48
3	8.61	7.03	15.64
Average	7.64	8.08	15.96

In Ehart *et al.* [13] the high strain region is calculated to a length of 5 mm, which is a bit lower than the numbers found in this experiment. The difference can be explained by the bending of the face layers and therefore a higher stress concentration in the core layer which leads to the shorter region of high strain, compared to the DCIB test setup.

## 4. Conclusions

In this work, the Double Cantilever I-beam test setup is analyzed in terms of the specific fracture energy and stress intensity factor as well as isotropic and orthotropic behavior by means of FE simulation. The FE simulation revealed a mostly similar process zone comparing orthotropic and isotropic stress and strain, which permits the usage of isotropic material characteristics for the calculation of the stress intensity factor K_I_.

Our own experiments of specific fracture energy and stress intensity factor calculation are compared to data of the wedge splitting experiment and numbers found in literature [13]. The stress intensity factor data is in the range of the data generated with the wedge splitting experiment. Furthermore it can be shown that elongated specimens reveal reduced data scattering. The data found for specific fracture energy is higher than the numbers found in literature and for the wedge splitting experiment. A probable explanation could be a multi layered crack on a micro basis as it appears with the internal bond strength test. The wedge splitting experiment, as well as the extended CT specimens are due to the bending of the face layers only impacted in the core layer and reveal therefore reduced specific fracture energy.

For the analysis of the crack length, it was supposed that the crack length would be longer when compared to standard test setups, due to the combination of elongated specimen geometry and ultra stiff braces in comparison to the stiffness of the specimen. Because of the low stiffness of the medium density fiberboards in tension perpendicularly to the board plane, a contactless full-field measurement system was used: electronic speckle pattern interferometry fulfilled all the requirements and could be shown to be appropriate. In addition to the strain analysis, the method made it possible to determine the crack length and the process zone size. Analyzing the strain in the direction normal to the board plane, an elongated process zone in plane direction was detected. Detailed analysis of the ESPI results showed a zone of high strain beyond the strain at failure estimated from internal bond strength and modulus of elasticity. In the high strained region fiber bridging takes places; the less strained region is subjected to micro cracking. The crack length measurements yielded a more elongated process zone than known from literature.

We conclude that the DCIB test setup shows low data scattering and is a powerful tool for the comparison of material characteristics in the core layer of wood based panels.
